# The Presence of a Physician Role Model and the Career Preference of Medical Students Are Associated With Rural Self-efficacy

**DOI:** 10.7759/cureus.46174

**Published:** 2023-09-29

**Authors:** Ryuichi Kawamoto, Asuka Kikuchi, Daisuke Ninomiya, Yoshio Tokumoto, Teru Kumagi

**Affiliations:** 1 Department of Community Medicine, Ehime University Graduate School of Medicine, Toon, JPN

**Keywords:** medical student, career preferences, physician role model, score, rural self-efficacy

## Abstract

Rural career preference is known to be affected by rural self-efficacy. This study aims to explore whether the presence of a physician role model and having a medical department of interest influence rural self-efficacy among medical students. The study sample comprised 813 students (464 male and 349 female). We assessed rural self-efficacy using a validated scale that comprised 15 questions. The effect of the presence of a physician role model and the choice of medical department on rural self-efficacy score was examined. Multivariable-adjusted regression analysis showed that the presence of a physician role model was significantly associated with the rural self-efficacy score (β = 0.236, *p *< 0.001), as were gender (β = −0.096, *p *= 0.004), admission while living in hometown (β = 0.077, *p *= 0.041), receiving a scholarship for regional duty (β = 0.079, *p *= 0.025), admission based on school recommendation (β = 0.077, *p *= 0.031), and subjective difficulty with living in a rural area (β = −0.201, *p *< 0.001). Moreover, a higher rural self-efficacy score was significantly associated with students who listed general medicine/family medicine (β = 0.204, *p *< 0.001), pediatrics (β = 0.098, *p *= 0.004), or obstetrics and gynecology (β = 0.108, *p *= 0.002) as their department of choice, while anesthesiology (β = −0.075, *p *= 0.023) was significantly associated with a lower rural self-efficacy score. These relationships were consistent for both males and females. The presence of a physician role model and the choice of medical department are important factors for higher rural self-efficacy scores.

## Introduction

Self-efficacy has been defined as an experience of confidence in one’s own worth and affirmation of one’s existence [1] or as a person’s belief in their ability to perform according to specific requirements [2]. Rural self-efficacy is associated with greater interest in rural medicine and career intentions in rural practice [3]. In recent years, rural self-efficacy has emerged as a concept that may influence individuals’ intentions regarding rural practice [3]. Four sources of individual self-efficacy have been proposed: mastery experiences, vicarious learning, social persuasion, and emotional and physical responses to experiences [1,4].

In Japan, this list should also include an assessment of one’s own specific preferences regarding work and the personal characteristics that are vital for working in healthcare in rural parts of the country. Our research [5] has included these aspects, unlike the investigations of other researchers [2,6]. Previous results from our study indicated that having a physician role model and a general medical orientation were significantly and independently associated with rural self-efficacy for women, while for men the important factors also included being in a lower academic year, graduating from a public high school, and not having failed an entrance exam [5]. Isaac et al. [3] demonstrated that female gender, rural background, rural clinical school preference for clinical training, and general practice intentions were factors associated with a higher level of self-efficacy.

Having a physician role model is an important factor influencing medical students’ values, attitudes, ethics, and professional behavior, and it plays a major role in their choice of future careers. In the medical field, a role model is a professional and experienced person whose actions make junior staff members subconsciously wish to emulate him or her [7]. Role models are critically important in the professional, character, and career development of students, and their presence effectively enhances the transformation of students into doctors [8].

Furthermore, the choice of medical department in the future may be an important factor in rural self-efficacy. General practice intentions are reported as a factor associated with higher levels of self-efficacy [3]. The significance of the "desire to practice in rural areas" plays a crucial role in selecting a career path in general/family medicine [9]. Aspiring medical students recognize that opting for general/family medicine as a profession is essential for those aiming to serve rural communities, where a diverse spectrum of medical conditions is expected. However, we are not aware of any reports examining the background of rural self-efficacy and considering its relationship with the choice of the medical department.

We, therefore, conducted this study as a cross-sectional survey of medical students in Japan, with the aim of investigating physician role models, the choice of medical department, and the effect of these factors on rural self-efficacy.

## Materials and methods

Participants

This study was designed as a cross-sectional study of students at Ehime University Graduate School of Medicine. All fifth-year medical students (approximately 100 students per year) from a local university medical school in Japan were recruited every year for nine years (2013-2021). During their orientation, before they commenced their clinical practice in a rural region, the students were given a questionnaire (in Japanese) and requested to answer all the questions in writing. Completing the survey was voluntary, and responses were anonymized. Participants with missing questionnaires were excluded from the analysis. The Institutional Review Board (IRB) of Ehime University Hospital (approval no. 1507004) reviewed and approved this study. All procedures were carried out in conformity with the applicable norms and regulations. All participants provided written informed consent.

Questionnaire

The questionnaire (survey questions aligned with the four sources of rural self-efficacy) we used to measure rural self-efficacy has previously been described and validated [5,10]. The questionnaire consisted of 15 questions and was developed to measure self-efficacy in rural practice. Self-efficacy was considered to arise from four potential sources: work preferences, evaluation of rural practices, rural living preferences, and the respondent’s personal character. In addition, the survey asked the participants to evaluate their own specific preferences regarding work and their personality traits that were crucial for working in healthcare in rural Japan, which was not included in previous studies [2,6,11]. All questions were scored using a Likert scale, and a composite score of rural self-efficacy was calculated for each respondent by summing all item scores. The lowest possible score was, therefore, 15, and the highest possible score was 60. Internal reliability, as indicated by Cronbach's alpha, was 0.849 for this sample [5]. As described above, the present survey included items necessary for evaluating self-efficacy [1], specifically adjusted for the evaluation of healthcare providers in rural areas.

The survey explored the participants’ background and their choice of medical department. The questions covered demographic characteristics, such as gender and age; the circumstances of the participants’ enrollment and schooling; whether they had passed or failed an entrance exam; whether their parents were physicians or whether they had other physician role models (questionnaire phrasing: "Did you encounter a physician whom you perceived as a role model?"); and whether they had been supported by a regional scholarship (“chiiki-waku” - the awarding of scholarships for regional duty is governed by a special policy with the principal aim of increasing the number of rural physicians), admitted based on school recommendation (a system whereby students can enter a medical school on the non-binding assumption that they will remain in a local prefecture and work there after graduation), or enrolled in another university. They were also asked about the size of the hometown they lived in until the age of 18. The categories were rural region, town, or village (population of 10,000-50,000), small city (population of 50,000-100,000), medium city (population of 100,000-500,000), and large city (population > 500,000). In addition, they were asked whether they felt that living in a rural region was difficult (rated on a Likert scale with four options: disagree = 1, slightly disagree = 2, slightly agree = 3, and strongly agree = 4).

Participants were asked to select the department they were pursuing from a total of 14 options: general/family medicine, internal medicine subspecialty, surgery, pediatrics, obstetrics/gynecology, psychiatry, anesthesiology, emergency medicine, dermatology, orthopedics, ophthalmology, otolaryngology, urology, and radiology, and “other” [12]. They were asked to specify which specialty was the first and second choice they were considering.

Statistical analyses

We used IBM Statistical Package for Social Science (SPSS) statistics v. 26 (IBM SPSS Statistics for Windows, Armonk, NY) for statistical analysis. A Kolmogorov-Smirnov test was performed for the normality of the sample data. If the data were normally distributed, we used the mean and standard deviation (SD) to express continuous variables; if not, we used the median (interquartile range). Differences based on the presence or absence of a physician role model were analyzed using chi-squared (χ^2^) tests or Mann-Whitney U tests. Correlations between various characteristics and rural self-efficacy scores were determined using Spearman’s rank correlation coefficient. Finally, we performed a multiple regression analysis to assess the contribution of confounding factors to the scores. Model A was adjusted using the forced entry method, and Model B was adjusted using the stepwise method. Both models included all variables, including the presence of a physician role model. Statistical significance was inferred at p < 0.05.

## Results

Participant characteristics with the presence of a physician role model

Table 1 presents the association of the characteristics of participating medical students with the presence of a physician role model. We received complete responses from 813 of 946 participants (85.9% response rate). Of these, 464 (57.1%) were male. The age range was 18-41 years, the median age was 22 years, and the interquartile range was 21-22 years. Students tended to have a physician role model significantly more frequently if they had a parent who was a doctor (p < 0.001), had not graduated from a public school (p = 0.046), or had not gained admission based on school recommendations (p = 0.029).

**Table 1:** Association of participant characteristics with the presence of a physician role model Data are presented as number (%). P-value from the χ^2^-test

Baseline characteristics (N = 813)		Physician role model		
		Absence (N = 472)	Presence (N = 341)	P-value
Gender	Female	193 (40.9)	156 (45.7)	0.173
	Male	279 (59.1)	185 (54.3)	
Age	< 21 years	220 (46.6)	153 (44.9)	0.669
	≥ 21 years	252 (53.4)	188 (55.1)	
Admission from Ehime prefecture where our university is located is located	No	227(48.1)	181 (53.1)	0.177
	Yes	245(51.9)	160 (46.9)	
Graduation from public high school	No	230 (48.7)	191 (56.0)	0.046
	Yes	242 (51.3)	150 (44.0)	
Graduation from junior high and high school	No	232 (49.2)	146 (42.8)	0.075
	Yes	240 (50.8)	195 (57.2)	
Had failed an entrance exam	No	263 (55.7)	168 (49.3)	0.075
	Yes	209 (44.3)	173 (50.7)	
Work experience	No	455 (96.8)	324 (95.0)	0.376
	Yes	17 (3.6)	17 (5.0)	
Experience with admission to another university	No	433 (91.7)	306 (89.7)	0.327
	Yes	39 (8.3)	35 (10.3)	
Had a parent who was a doctor	No	370 (78.4)	203 (59.5)	< 0.001
	Yes	102 (21.6)	138 (40.5)	
Scholarship for regional duty	No	363 (76.9)	255 (74.8)	0.506
	Yes	109 (23.1)	86 (25.2)	
Admission based on school recommendation	No	319 (67.6)	255 (74.8)	0.029
	Yes	153 (32.4)	86 (25.2)	
Size of hometown of residence until 18 years of age	Small, medium, or large city	414 (87.7)	302 (88.6)	0.743
	Rural, remote, town, or village	58 (12.3)	39 (11.4)	
Living in a rural area is challenging	Disagree or strongly disagree	274 (58.1)	221 (64.8)	0.058
	Agree or strongly agree	198 (41.9)	120 (35.2)	

Association of career preferences with the presence of a physician role model

Table 2 presents the association between medical students’ choice of medical department and the presence of a physician role model. Students who chose pediatrics (p < 0.001), obstetrics and gynecology (p = 0.027), and emergency medicine (p = 0.005) were significantly more likely to have had a physical role model, but those who chose otolaryngology (p = 0.016) as a career were less likely to have had such a role model. There was no such significant association with the choice of general/family medicine, special internal medicine, surgery, or other specialties as a career.

**Table 2:** Association of medical students’ career preferences with the presence of a physician role model 1^st^ + 2^nd^, first and second choice of career preferences Data are presented as number (%). P-value from the χ^2^-test.

		Physician role model		
Specialty preference (N = 813)	Choice	Absence (N = 472)	Presence (N = 341)	P-value
General/family medicine	1^st ^+ 2^nd^	273 (57.8)	205 (60.1)	0.564
Special internal medicine	1^st ^+ 2^nd^	171 (36.2)	108 (31.7)	0.179
Surgery	1^st ^+ 2^nd^	181 (38.3)	128 (37.5)	0.826
Pediatrics	1^st ^+ 2^nd^	129 (27.3)	134 (39.3)	< 0.001
Obstetrics & gynecology	1^st ^+ 2^nd^	73 (15.5)	74 (21.7)	0.027
Psychology	1^st ^+ 2^nd^	93 (19.7)	66 (19.4)	0.929
Anesthesiology	1^st ^+ 2^nd^	86 (18.2)	65 (19.1)	0.784
Emergency medicine	1^st ^+ 2^nd^	83 (17.6)	88 (25.8)	0.005
Dermatology	1^st ^+ 2^nd^	69 (14.6)	40 (11.7)	0.252
Orthopedics	1^st ^+ 2^nd^	92 (19.5)	57 (16.7)	0.358
Ophthalmology	1^st ^+ 2^nd^	64 (13.6)	40 (11.7)	0.458
Otolaryngology	1^st ^+ 2^nd^	51 (10.8)	20 (5.9)	0.016
Urology	1^st ^+ 2^nd^	18 (3.8)	13 (3.8)	1.000
Radiology	1^st ^+ 2^nd^	46 (9.7)	31 (9.1)	0.809
Others	1^st ^+ 2^nd^	15 (3.2)	19 (5.6)	0.110

Association of sources of the rural self-efficacy score with the presence of a physician role model

**Figure 1:**
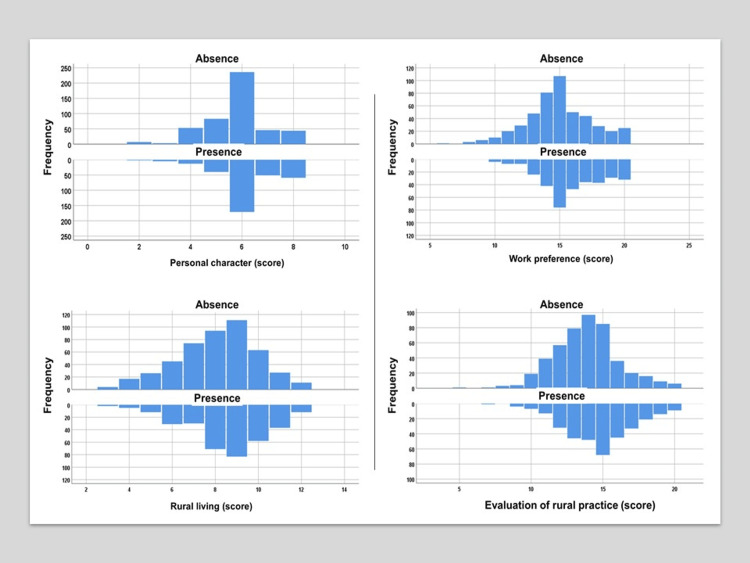
Distribution of four sources of the rural self-efficacy score by a physician role model A Kolmogorov-Smirnov test was performed for the normality of the sample data (p < 0.001). The data were not normally distributed.

**Figure 2:**
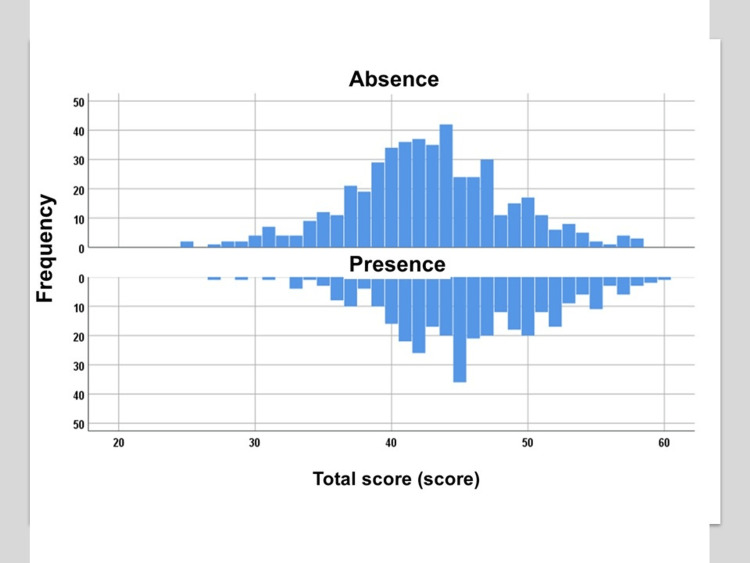
Distribution of the rural self-efficacy score by a physician role model A Kolmogorov-Smirnov test was performed for the normality of the sample data (p = 0.002). The data were not normally distributed.

Figures 1-2 show the scores and distribution of four sources that constitute rural self-efficacy, stratified by the presence or absence of a physician role model. As shown in Table 3, those who had a physician role model had significantly higher scores for all four sources (p < 0.001 for all).

**Table 3:** Four sources of rural self-efficacy score by a physician role model Data for rural self-efficacy were skewed and, thus, presented as median (interquartile range) values. P-value from the Mann-Whitney U test

Four sources of the rural self-efficacy score (N = 813)	A physician role model		
	Absence (N=472)	Presence (N=341)	P-value
Work preference (range, 5-20)	15.0 (14.0-16.0)	16.0 (15.0-18.0)	< 0.001
Evaluation of rural practice (range, 5-20)	14.0 (12.0-15.0)	15.0 (13.0-16.0)	< 0.001
Rural living (range, 3-12)	8.0 (7.0–9.0)	9.0 (8.0-10.)	< 0.001
Personal character (range, 2- 8)	6.0 (5–6)	6.0 (6.0-7.0)	< 0.001
Total score (range, 15 - 60)	43.0 (39.0–46.0)	45.0 (42.0-50.0)	< 0.001

Relationship between characteristics, including the presence of a physician role model and rural self-efficacy score

According to the simple (non-adjusted) model, the presence of a physician role model (r_s_ = 0.245, p < 0.001) and admission while living in their hometown (r_s_ = 0.137, p < 0.001), graduation from public high school (r_s_ = 0.087, p = 0.014), graduation from junior high and high school (r_s_ = -0.073, p = 0.037), receiving a scholarship for regional duty (r_s_ = 0.125, p < 0.001), admission based on school recommendation (r_s_ = 0.130, p < 0.001), size of hometown of residence until the age of 18 (r_s_ = 0.090, p = 0.011), and subjective difficulty with living a rural area (r_s_ = -0.227, p < 0.001) were significantly associated with the rural self-efficacy score (Table 4). Multivariable-adjusted regression model B (stepwise parameter entry) showed that the presence of a physician role model (β = 0.236, p < 0.001) was significantly associated with a rural self-efficacy score, as were gender (β = -0.096, p = 0.004), admission while living in hometown (β = 0.077, p = 0.041), receiving a scholarship for regional duty (β = 0.079, p = 0.025), admission based on school recommendation (β = 0.077, p = 0.031), and subjective difficulty with living in a rural area (β = -0.201, p < 0.001).

**Table 4:** Relationship between characteristics including the presence of a physician role model and rural self-efficacy score Model A, multivariable-adjusted for all variables; Model B, stepwise method; β, standard regression coefficient

	Rural self-efficacy score		
	Non -adjusted	Multivariable-adjusted	
		Model A	Model B
Characteristics (N = 813)	Spearman’s r_s_ (p-value)	β (p-value)	β (p-value)
Gender: Female = 0, Male = 1	－0.094 (0.007)	－0.106 (0.002)	－0.096 (0.004)
Age: < 21 years =0, ≥ 21 years = 1	－0.063 (0.073)	－0.046 (0.278)	-------
Admission while living in hometown: No = 0, Yes = 1	0.137 (< 0.001)	0.063 (0.107)	0.077 (0.041)
Graduation from public high school: No = 0, Yes = 1	0.087 (0.014)	0.002 (0.973)	-------
Graduation from junior high and high school: No = 0, Yes = 1	－0.073 (0.037)	－0.022 (0.712)	-------
Has failed entrance exam: No = 0, Yes = 1	－0.058 (0.099)	－0.014 (0.751)	-------
Work experience: No = 0, Yes = 1	0.009 (0.787)	0.020 (0.637)	-------
Experience with admission to another university: No = 0, Yes = 1	－0.024 (0.492)	0.015 (0.718)	-------
Has a parent who is a doctor: No = 0, Yes = 1	0.026 (0.456)	0.003 (0.926)	-------
Scholarship for regional duty: No = 0, Yes = 1	0.125 (< 0.001)	0.080 (0.029)	0.079 (0.025)
Admission by school recommendation: No = 0, Yes = 1	0.130 (< 0.001)	0.061 (0.119)	0.077 (0.031)
Hometown of residence size until 18 years of age: Small, medium, or large city = 0, Rural, remote, town, or village = 1	0.090 (0.011)	0.051 (0.138)	-------
Difficulty with living in a rural area: Disagree or strongly disagree = 0, Agree or Strongly agree = 1	－0.227 (< 0.001)	－0.194 (< 0.001)	－0.201 (< 0.001)
A physician role model: Absence = 0, Presence = 1	0.245 (< 0.001)	0.237 (< 0.001)	0.236 (< 0.001)
R^2^	-------	0.189 (< 0.001)	0.141 (< 0.001)

Relationship between career preferences and rural self-efficacy score

A specialty preference for general/family medicine (β = 0.204, p < 0.001), pediatrics (β = 0.098, p = 0.004), or obstetrics and gynecology (β = 0.108, p = 0.002) was significantly associated with a higher rural self-efficacy score, even after adjusting for significantly related factors. However, a preference for anesthesiology (β = -0.075, p = 0.023) was significantly associated with a lower rural self-efficacy score (Table 5).

**Table 5:** Relationship between medical students' career preferences and rural self-efficacy score 1st + 2nd, first and second choice of career preferences; Model, adjusted for all variables, which were significant in Model B in Table 4; β, standard regression coefficient

		Rural self-efficacy score	
Specialty preference (N=813)	Choice	β	P-value
General medicine/Family medicine	1^st ^+ 2^nd^	0.204	< 0.001
Special internal medicine	1^st ^+ 2^nd^	0.004	0.898
Surgery	1^st ^+ 2^nd^	0.006	0.862
Pediatrics	1^st ^+ 2^nd^	0.098	0.004
Obstetrics & gynecology	1^st ^+ 2^nd^	0.108	0.002
Psychology	1^st ^+ 2^nd^	0.043	0.184
Anesthesiology	1^st ^+ 2^nd^	－0.075	0.023
Emergency medicine	1^st ^+ 2^nd^	－0.018	0.583
Dermatology	1^st ^+ 2^nd^	－0.036	0.289
Orthopedics	1^st ^+ 2^nd^	－0.004	0.905
Ophthalmology	1^st ^+ 2^nd^	－0.045	0.191
Otolaryngology	1^st ^+ 2^nd^	－0.012	0.729
Urology	1^st ^+ 2^nd^	0.044	0.173
Radiology	1^st ^+ 2^nd^	－0.006	0.850
Others	1^st ^+ 2^nd^	－0.013	0.677

Relationship between all characteristics and rural self-efficacy score by gender

Table 6 shows the medical students stratified by gender. Consistent with previous findings, the presence of a role model was significantly and independently associated with rural self-efficacy for both males and females. Furthermore, general practice and pediatric aspirations were associated with higher self-efficacy scores for males, and obstetrics and gynecology aspirations were associated with higher self-efficacy scores for females.

**Table 6:** Relationship between all characteristics and rural self-efficacy score by gender β, standard regression coefficient; Model, adjusted for all variables, which were significant in Model B in Table 4 and Table 5

	Rural self-efficacy score	
	Multivariable-adjusted	
	Male	Female
Baseline characteristics (N = 813)	β (p-value)	β (p-value)
Admission while living in hometown: No = 0, Yes = 1	0.063 (0.191)	0.108 (0.050)
Scholarship for regional duty: No = 0, Yes = 1	0.089 (0.050)	0.008 (0.876)
Admission by school recommendation: No = 0, Yes = 1	0.080 (0.086)	0.045 (0.381)
Difficulty with living in a rural area: Disagree or strongly disagree = 0, Agree or Strongly agree = 1	－0.157 (< 0.001)	－0.233 (< 0.001)
A physician role model: Absence = 0, Presence = 1	0.266 (< 0.001)	0.150 (0.002)
General medicine/Family medicine	0.161 (< 0.001)	0.259 (< 0.001)
Pediatrics	0.130 (0.003)	0.064 (0.204)
Obstetrics & gynecology	0.027 (0.534)	0.158 (0.002)
Anesthesiology	－0.050 (0.240)	－0.117 (0.015)
R^2^	0.197 (< 0.001)	0.236 (< 0.001)

## Discussion

The present study examined the factors associated with rural self-efficacy among medical students in Japan. We found that the presence of a physician role model and the specific preferences of medical students’ careers were significantly associated with a higher score for rural self-efficacy, as were gender, admission while living in a hometown, receiving a scholarship for regional duty, admission based on school recommendation, and subjective difficulty with living in a rural area. These relationships were consistent for both males and females. To the best of our knowledge, no previous report has clarified the factors associated with rural self-efficacy among Japanese medical students.

The scale we developed is structured around an interest in rural practice but is also consistent with self-efficacy. For example, the components of Factor 2 of “There are many opportunities in rural areas that can improve one’s career,” “Working in a rural area provides more opportunities to practice a variety of skills,” and ”Rural practice provides greater opportunities for work autonomy” would correspond to mastery experiences that strongly and consistently affect self-efficacy. Components of Factor 1 of “I would like to be concerned with a patient's life throughout treatment,” “I would like to support the patient’s welfare,” “I want to be a doctor who accompanies the patient and works with the patient on their problems,“ and “I want to be a doctor who accompanies the patient and works with the patient on their problems” correspond to vicarious learning, which is triggered by observing others' actions. Factor 2 component “Possibilities are felt for community medicine in rural areas” corresponds to a social persuasion. Factors 3 and 4 correspond to emotional and physiological reactions. However, while this score has been tested for its relationship to students' future intent to engage in rural practice, it has not been tested for its association with actual behavior [5]. Reports from Australia indicate that rural self-efficacy has an independent association with willingness to remain in or return to a small rural practice [2]. Perceiving living in a rural area as challenging reduced future career intentions in rural areas, but a higher level of rural self-efficacy ameliorated this relationship [10]. Moreover, although social isolation during clinical practice in rural areas is commonly reported and has been shown to reduce the inclination to work in rural areas, having a higher level of rural self-efficacy means that social isolation is less likely to affect a person’s future intentions regarding rural living [13]. In other words, self-efficacy plays a role as a protective factor against one of the components of burnout in rural physicians. Therefore, special attention should be paid to improving self-efficacy as an important part of burnout-prevention programs for rural physicians [14]. Rural self-efficacy may, thus, be a meaningful conceptual construct in explaining rural career intentions.

The effect of the existence of role models has been the subject of numerous reports in the field of medical education. In the present study, we found role models to be an explanatory factor for rural self-efficacy. In reports examining the characteristics of the role models themselves, the most frequently cited characteristics were as follows: as physicians, enthusiasm for the specialty, clinical reasoning skills, seeing the patient as a whole, and doctor-patient relationship; as teachers, enthusiasm for teaching, student involvement, and effective communication with students; and as people, enthusiasm, compassion, and competence [15]. Fortunately, most human behavior can be learned through observation. By observing others, we form ideas about how new behaviors are performed, and later this coded information guides our own behavior [16]. For the medical students in this study, the presence of a physician role model was an important independent factor associated with rural self-efficacy. Our interpretation is that the students learned concepts that boosted their rural self-efficacy from the ideas and enthusiasm for the work of their role models. In other words, the rural self-efficacy score we developed may be representative of the effect of the presence of a physician role model [15]. It appears that a small number of medical students may be influenced by such an image of the physician.

A preference for general internal/family medicine, pediatrics, or obstetrics and gynecology was significantly associated with a higher rural self-efficacy score, even after adjusting for various background factors. All these specialties are considered essential in rural and remote areas and are also associated with a specific type of uncontrollable lifestyle [17]. Rural practice activities require a wide range of responses from a limited number of physicians. Medical students who wish to major in these medical specialties are also willing to engage in a wide range of activities, which may contribute to their higher level of rural self-efficacy.

We found that admission while living in a hometown, receiving a scholarship for regional duty, admission based on school recommendation, and perceiving living in rural regions as challenging were independently and significantly associated with the score for rural self-efficacy. Admission based on school recommendation and the scholarship for regional duty is governed by a special policy with the principal aim of increasing the number of local physicians [18,19]. All of these factors are closely related to local community healthcare and were found to be relevant to rural self-efficacy. The perception of living in rural regions as challenging was strongly influenced by personal connections (e.g., family background and personal experiences), which resulted in positive or negative associations with rural life and also factored into the decision on whether to live in rural areas [20]. This perception had a negative effect on rural self-efficacy and even on the wish to pursue a rural career [13,20].

The quantitative approach we used to assess the effects of rural self-efficacy was an important strength of this study. The study also had a large sample size. Another strength was that we were able to make adjustments for potential confounding factors. However, the study was also subject to some limitations. Our cross-sectional study design does not eliminate potential causal relationships between the presence of a physician role model and the career preferences of medical students and rural self-efficacy. In addition, because the study was restricted to one educational institution in a rural area, the generalizability of our findings may be limited. Finally, we assessed only the students’ intentions to study in particular medical departments, rather than their actual choices.

## Conclusions

The presence of a physician role model and specific departments of interest (e.g., general/family medicine, pediatrics, or obstetrics/gynecology) were significantly associated with higher rural self-efficacy scores. We, therefore, suggest that opportunities to meet a physician role model and learn about their specialty may be important for fostering the development of rural self-efficacy in Japan’s medical community.

## Appendices

**Table 7:** Questionnaires: Survey questions aligned with the four sources of rural self-efficacy The score is obtained by summing the scores for all the items, with the lowest possible score being 15 and the highest being 60.

Sources of rural self-efficacy
Questions: Strongly disagree = 1, slightly disagree = 2, slightly agree = 3, strongly agree = 4
Factor 1: Work preferences
I would like to be concerned about a patient’s life throughout their treatment.
I would like to support the patient’s welfare.
I want to be a doctor who accompanies the patient and works with them on their problems.
I would like to provide continuous care for the patient from an early stage.
I am interested in the patients themselves (e.g., children and older adults).
Factor 2: Evaluation of rural practice
There are many opportunities in rural areas that could improve my career.
Working in a rural area provides more opportunities to practice a variety of skills.
I see possibilities for community medicine in rural areas.
I am interested in research activities in the rural field.
Rural practice provides greater opportunities for work autonomy.
Factor 3: Rural living preferences
Living in rural areas does not bother me.
I would like to bring up a child in a rural area.
There are things I enjoy doing in rural areas.
Factor 4: Personal character
I like to talk with people.
I like to talk with medical colleagues (e.g., nurses).
